# Revealing the Potential Markers of N(4)-Acetylcytidine through acRIP-seq in Triple-Negative Breast Cancer

**DOI:** 10.3390/genes13122400

**Published:** 2022-12-18

**Authors:** Xingda Zhang, Jiaqi Zeng, Jianyu Wang, Zihan Yang, Song Gao, Honghao Liu, Guozheng Li, Xin Zhang, Yue Gu, Da Pang

**Affiliations:** 1 Department of Breast Surgery, Harbin Medical University Cancer Hospital,150 Haping Road, Harbin 150081, China; 2 School of Life Science and Technology, Computational Biology Research Center, Harbin Institute of Technology, Harbin 150001, China

**Keywords:** N(4)-acetylcytidine (ac4C), TNBC, acRIP-seq, prognostic risk marker, drug target prediction

## Abstract

Understanding the causes of tumorigenesis and progression in triple-receptor negative breast cancer (TNBC) can help the design of novel and personalized therapies and prognostic assessments. Abnormal RNA modification is a recently discovered process in TNBC development. TNBC samples from The Cancer Genome Atlas database were categorized according to the expression level of NAT10, which drives acetylation of cytidine in RNA to N(4)-acetylcytidine (ac4C) and affects mRNA stability. A total of 703 differentially expressed long non-coding RNAs (lncRNAs) were found between high- and low-expressed NAT10 groups in TNBC. Twenty of these lncRNAs were significantly associated with prognosis. Two breast cancer tissues and their paired normal tissues were sequenced at the whole genome level using acetylated RNA immunoprecipitation sequencing (acRIP-seq) technology to identify acetylation features in TNBC, and 180 genes were significantly differentially ac4c acetylated in patients. We also analyzed the genome-wide lncRNA expression profile and constructed a co-expression network, containing 116 ac4C genes and 1080 lncRNAs. Three of these lncRNAs were prognostic risk lncRNAs affected by NAT10 and contained in the network. The corresponding reciprocal pairs were “LINC01614-COL3A1”, “OIP5-AS1-USP8”, and “RP5-908M14.9-TRIR”. These results indicate that RNA ac4c acetylation involves lncRNAs and affects the tumor process and prognosis of TNBC. This will aid the prediction of drug targets and drug sensitivity.

## 1. Introduction

Breast cancer (BC) is a complex heterogeneous disease with a globally increasing incidence [1]. Triple-receptor negative BC (TNBC) is an aggressive form of BC in which cells do not express ER, progesterone receptors, or HER2 and for which treatments are ineffective. Several molecular targets are being explored to target TNBC, including the androgen receptor, epidermal growth factor receptor (EGFR), poly (ADP-ribose) polymerase (PARP), and vascular endothelial growth factor (VEGF) [2] TNBC accounts for approximately 15–20% of BC. In contrast to hormone receptor-positive or HER2-positive disease, TNBC has a highly aggressive clinical course, earlier age of onset, greater metastatic potential, and worse clinical outcome, as evidenced by higher recurrence rates and lower survival rates [3].

N4-acetylcytidine (ac4C) is generally considered a conserved, chemically modified nucleoside of RNA. However, recent studies have found extensive ac4C modifications in human and yeast mRNAs that affect translation efficiency and mRNA stability [4]. Studies by Thomale et al. [5] and Liebich et al. [6] found a significant increase in modified nucleosides (including ac4C) in the urine of tumor-bearing mice and cancer patients. Most importantly, recent studies have shown that levels of modified nucleosides, such as ac4C, are frequently altered in urine samples from patients with multiple cancers [7,8,9]. Compared with the control group, ac4C levels in urine samples of BC patients were significantly increased, and area under the curve receiver operator characteristic (AUC-ROC) analysis of ac4C was 0.825 in BC diagnosis [9]. Several other studies have also shown that ac4C levels are associated with inflammatory responses [10,11]. These findings indicate that ac4C is a potential biomarker for cancer and is important in the diagnosis and treatment of cancer.

NAT10 (also known as hALP, human N-acetyltransferase-like protein), has histone acetylation activity and was the first acetylation regulator shown to maintain efficient translation and stabilization of mRNA by forming ac4C on mRNA [12]. Duan et al. showed that NAT10 is associated with cancer by significantly promoting cell growth in epithelial ovarian cancer and BC [13,14]. In addition, NAT10 may increase melanogenesis and melanoma growth [15]. These findings indicate a multifaceted functional role for NAT10 in cancer. A total of 9 of the 33 cancer types in The Cancer Genome Atlas (TCGA) database show a significant relationship between NAT10 expression levels and overall survival (OS); however, 7 show a significant association with progression-free survival (PFS), 5 with disease free survival (DFS), and 7 with disease special survival (DSS). Overall, NAT10 expression is an independent risk factor for poor prognosis in these cancers [16].

Long non-coding RNAs (lncRNAs) are endogenous single-stranded RNAs ranging from 200 bp to 1000 kb that do not encode proteins [17]. lncRNAs can regulate DNA methylation, histone modification, and chromosome remodeling through epigenetic, transcriptional, and post-transcriptional regulation [18]. As a new class of biomarkers, lncRNAs have been widely studied in the pathogenesis, diagnosis, and drug screening of various diseases [19]. Their dysregulation and abnormal expression patterns are associated with various cancer types, including TNBC [20,21]. The lncRNA, SNHG14, induces BC resistance to trastuzumab through H3K27 acetylation-mediated regulation of PABPC1 expression, lncRNA GHSROS significantly promotes growth and migration of BC, and lncRNA NONHSAT101069 regulates Twist1 by targeting the microRNA, miR-129-5p, to induce epirubicin resistance and promote BC cell migration and invasion [22]. In addition, a novel lncRNA named RP11-22N19.2 was highly overexpressed in TNBC compared with non-TNBC tissues, which predicted poor prognosis for overall survival and recurrence-free survival in TNBC [23]. In conclusion, lncRNAs play a key role in the development of breast cancer.

The prognosis of TNBC patients is greatly affected by the absence of key biomarkers and drug targets. Ac4C plays a key role in transcription and translation and disruption of its regulator, NAT10, is closely related to the occurrence and prognosis of various cancers. In this study, we combined TCGA data with data from acRIP-seq sequencing to identify lncRNAs involved in RNA ac4C acetylation and TNBC development. These lncRNAs are significantly related to the prognosis of TNBC patients, and can inform the development of TNBC-specific drug targets and chemosensitivity prediction.

## 2. Materials and Methods

### 2.1. Detection and Quantification of ac4C in Clinical Samples

This study was approved by the Ethics Committee of Harbin Medical University, and written informed consent was obtained from all participants prior to inclusion. Tumor and paracancerous samples were surgically obtained from two patients with TNBC at Harbin Medical University Cancer Hospital. Neither patient had received prior neoadjuvant or other therapy.

After adding 1 mL of TRIzol™ Reagent per 50–100 mg of tissue, samples were disrupted by high-frequency reciprocating vibration with grinding beads on ice, and then the total RNA was isolated and purified by the phenol–chloroform method. After digesting DNA in 150 µg total RNA samples, an Epi^TM^ ac4C immunoprecipitation kit was used to perform RNA fragmentation according to the manufacturer’s instructions. Zymo RNA clean and a concentrator-25 kit were used to purify and recover the fragmented RNA. An anti-N4-acetylcytidine (ac4C) antibody was used to immunoprecipitate ac4C-modified RNA, which was recovered using a HiPure cell miRNA Kit after washing with high salt buffer. Libraries were prepared using the Epi^TM^ mini longRNA-seq kit, and the Bioptic Qsep100 Analyzer was used to inspect library quality to check whether the library size distribution conformed to the theoretical size. Finally, the NovaSeq high-throughput sequencing platform in PE150 sequencing mode was used to sequence the TNBC and paracancerous libraries.

### 2.2. Analysis of acRIP-seq and Peak Calling

Total RNA before and after treatment was sequenced by acRIP-seq, and the sequence data were obtained by base identification and error filtering. The quality of the sequencing data was analyzed by fastQC to determine sequencing quality distribution, base content distribution, and repeat sequencing fragment ratio. The clean data were obtained by de-junctioning and quality control of the raw data for subsequent analysis. The clean reads were then compared with the human reference genome hg38 using HISAT2 software to obtain unique mapped reads for the next step of analysis. exomePeak was used for peak calling. Finally, we used HOMER (http://homer.ucsd.edu/homer/ngs/peakMotifs.html (accessed on 12 May 2021)) software to perform ac4C motif analysis on peaks.

### 2.3. Difference Analysis of ac4C Modification

By finding differences between samples or between disease groups (treatment groups) and controls, we can obtain the differential ac4C modification levels for a particular disease (or treatment conditions) and thus explain the role of the episomal transcriptome in disease onset and progression at the ac4C level. In this paper, we use the exomePeak R package to reveal the dynamics of post-transcriptional regulatory RNA acetylation in two sequencing experiments. The differential analysis was performed in 2 steps. Firstly, peaks detection, and then the presence or absence of differential acetylation modification (statistical test) of these sites. The differential multiplicity method was used to calculate the degree of difference. The genes that were significantly differentially acetylated were screened for subsequent analysis using *p* < 0.05 as the screening criterion.

### 2.4. Functional Enrichment Analysis

A functional enrichment analysis was carried out on ac4C genes. The Gene Ontology (GO) database is a comprehensive database describing gene functions, divided into three parts: molecular functions, biological processes, and cellular components. The Kyoto Encyclopedia of Genes and Genomes (KEGG) is a comprehensive database integrating genomic, chemical, and systemic functional information. Different genes coordinate with each other to perform their functions, and significant enrichment by pathway analysis allows the identification of the most important biochemical metabolic pathways and signal transduction pathways associated with candidate target genes. The identified differential ac4C genes were functionally annotated at GO and KEGG levels based on the DAVID database (https://david.ncifcrf.gov/ (accessed on 15 August 2021)), and the significance level (*p*-value) of each GO term was calculated using Fisher’s test to screen out the pathways and functions with significance when *p.adj* < 0.01.

### 2.5. Expression of Differentially Expressed Genes Affected by NAT10 and Assessment of Immune Cell Infiltration in the TCGA Breast Cancer Cohort

To further explore how NAT10 influences the development of TNBC, we obtained RNA-seq data for TNBC from the TCGA database (https://portal.gdc.cancer.gov/ (accessed on 1 July 2021)). Gene expression information from 116 TNBC patient samples was included. These samples were divided into two groups based on the median expression level of NAT10: high NAT10 and low NAT10. The limma package [24] was used to screen for differentially expressed lncRNAs between the two groups (*p* < 0.05 and |FC| > 2). We also assessed immune cell infiltration in TCGA TNBC samples using ImmuCellAI (http://bioinfo.life.hust.edu.cn/ImmuCellAI (accessed on 20 July 2021)) to explore differences in the proportion of immune cell infiltration between different clusters.

### 2.6. Merging of Differential ac4C Genes

In this study, acRIP-seq data from different public platforms were integrated using the robust rank aggregation (RRA) method to obtain the integrated differential ac4C genes from different platforms. The RRA method uses a probabilistic model for aggregation, which is robust to noise and helps to calculate the probability of occurrence of significance for all genes in the final ranking. Robust Rank Aggregation is another R package that mainly combines the results of variance analysis from different platforms with the Robust Rank Aggregation (RRA) algorithm to obtain a comprehensive list of variance significance rankings. The different sets of platform variance genes are intersected while also considering their ranking. Overall, the genes that showed differences in multiple datasets and ranked high for each difference were selected after the final difference gene merge. We finally selected genes with significant differences (*p* < 0.05) in the integrated dataset, and genes differentially modified in both sequencing as the final selection of ac4C differential genes.

### 2.7. Constructing a Network to Screen for ac4C-Related Prognostic Risk lncRNAs

To screen ac4C-related prognostic risk lncRNAs, this study used Pearson correlation analysis to construct a co-expression network of lncRNAs and ac4C differential genes. In the TCGA database, lncRNAs with significantly different expression between TNBC and normal groups were screened by *t*-test (*p.adj* < 0.05). Pearson correlation analysis was performed on the expression levels of differential ac4C genes and differentially expressed lncRNAs in TNBC samples, and the co-expression network was constructed to screen for significant ac4C gene -lncRNA correlations (R > 0.5, *p* < 0.05). One-step neighbor lncRNAs of ac4C prognostic risk genes were mined in the co-expression network, and these lncRNAs were intersected with the list of prognostic risk lncRNAs affected by NAT10 to obtain ac4C-related prognostic risk lncRNAs, as well as their co-expressed ac4C genes.

### 2.8. Prediction of Drug Targets and Sensitivity

Data related to drugs and their drug targets were obtained from the DRUGBANK database and intersected with ac4C differential genes to construct a drug-target network from which drug targets for prognostic risk ac4C differential genes were mined. There is a lack of efficacious target drugs for TNBC; therefore, we used the ac4C-related lncRNA signature to perform drug sensitivity prediction using R package “pRRophetic” [25]. With reference to the latest TNBC dosing consensus, doxorubicin and paclitaxel were selected for drug sensitivity prediction [26]. From the pharmacogenomics database “The Genomics of Drug Sensitivity in Cancer” (GDSC) (https://www.cancerrxgene.org/ (accessed on 10 January 2022)) [27], the half-maximal inhibitory concentration (IC50) of TCGA samples was estimated by ridge regression against the GDSC training set [28]. Tenfold cross-validation was used to evaluate prediction accuracy of the estimated IC50. The Mann–Whitney–Wilcoxon test was used to determine whether IC50 distributions of high-risk and low-risk groups were identical.

## 3. Results

### 3.1. Workflow

**Figure 1:**
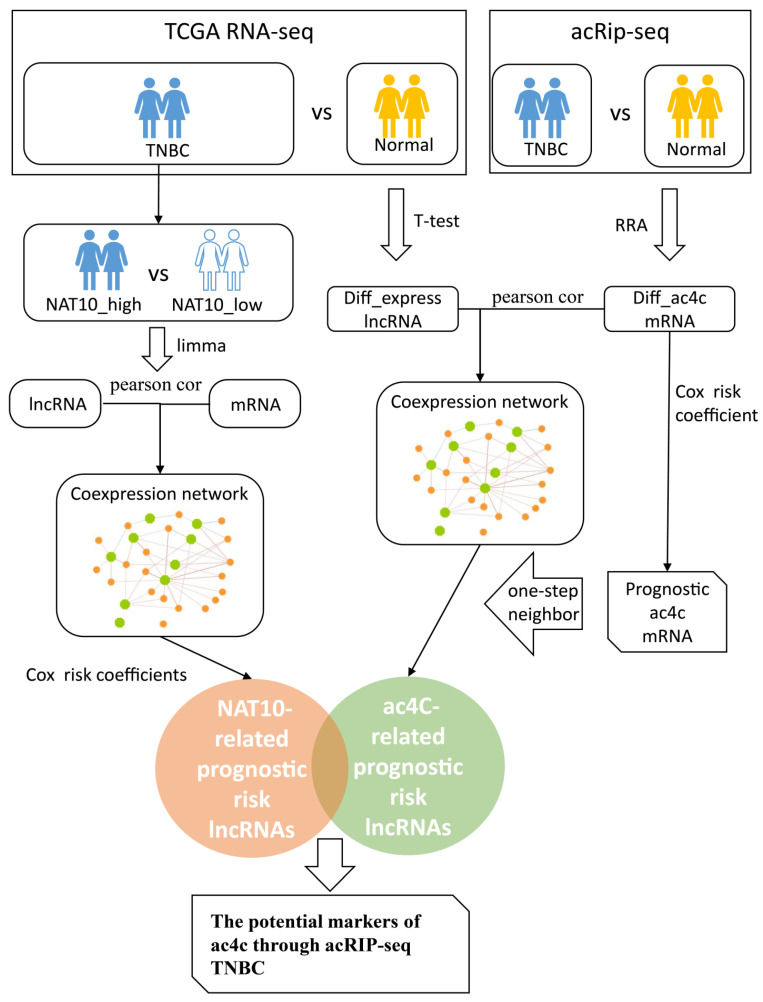
The study workflow.

The study design is presented in Figure 1.

### 3.2. Assessment of NAT10 Characteristics in TNBC Based on TCGA Database

**Figure 2:**
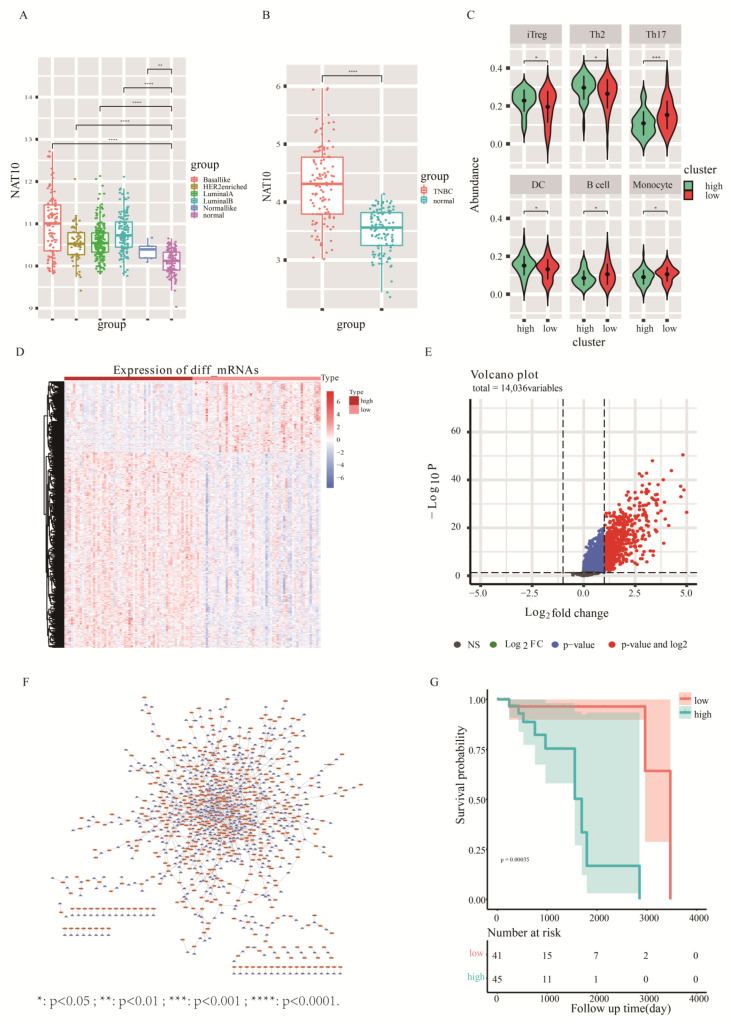
Assessment of NAT10 characteristics in TNBC based on the TCGA database. (**A**) Expression levels of NAT10 in various molecular subtypes of TCGA breast cancer. (**B**) Expression levels of NAT10 in TNBC and paraneoplastic samples. (**C**) Differences in the abundance of immune cell infiltration in samples from high- and low-NAT10 expression groups. (**D**) Heatmap of differentially expressed genes (mRNA levels) between high- and low-NAT10 expression groups. (**E**) Volcano plot of the analysis of differentially expressed lncRNAs in high- and low-NAT10 expression groups. (**F**) Co-expression network of lncRNAs and mRNAs showing differences between NAT10 high- and low-groups. The blue triangle nodes are mRNAs, and the orange circle nodes are lncRNAs. (**G**):The survivel analysis between high-risk and low-risk group. *: *p* < 0.05 ; **: *p* < 0.01 ; ***: *p* < 0.001 ; ****: *p* < 0.0001.

RNA-seq data of breast cancer patients (1104 cases) with cancer tissues and (113 cases) with paracancer tissues were obtained from the TCGA database. The expression levels of NAT10 in breast cancer were annotated based on sequencing reads of NAT10. The comparison revealed that NAT10 expression was significantly higher in all PAM50 subtypes of breast cancer compared to paraneoplastic tissues. Among them, the basal-like subtype showed the most significant upregulation (Figure 2A, *t*-test, *p* < 0.05). The similar significant upregulation in the expression of NAT10 was also found in 116 TNBC cases (Figure 2B, *t*-test, *p* < 0.05). Further, the present study carried out an in depth exploration of TNBC.

There is a recognized correlation between tumorigenesis and the immune microenvironment; therefore, we investigated the relationship between NAT10 expression and immune cell infiltration in TNBC using the immune cell abundance prediction database, ImmuCellAI [29], to predict TNBC immune cell abundance. TNBC samples were divided into high- and low-expression groups using NAT10 expression. Box plots of differences in immune cell infiltration between high- and low-expression groups showed that the infiltration of immune cells, such as iTreg, Th2, Th17, DC, B cells, and monocytes, in TNBC was related to NAT10 expression (Figure 2C, *t* test, *p* < 0.05). The numbers of iTreg, Th2, and DC cells were higher in the high-NAT10 expression group, and the numbers of Th17, B cells, and monocytes were higher in the low-NAT10 expression group than the other group. These results indicated that NAT10 is involved in the pathogenesis of TNBC by affecting the tumor immune microenvironment.

Changes in mRNA and lncRNA levels between high- and low-NAT10 expression groups in TNBC were also observed. The 731 significantly upregulated mRNAs (Figure 2D) and 730 significantly differentially upregulated lncRNAs (*p.adj* < 0.05&FC > 2) were screened using the limma package (Figure 2E). Pearson correlation analysis of expression between 730 differentially expressed lncRNAs and 731 differentially expressed mRNAs was performed revealing 1157 pairs of relationships containing 470 lncRNAs and 591 mRNAs (r > 0.5) (Figure 2F). The expression of these 470 lncRNAs was significantly different between high- and low-NAT10 expression groups in TNBC.

To determine the most prognostically relevant lncRNA features, single-factor Cox and multi-factor Cox regression analyses were carried out by combining age, gender, stage, TNM stage, and other clinical factors of TNBC patients. Finally, 12 lncRNAs that were significantly associated with prognosis were identified (*p* < 0.05). The sum of the product of their multi-factor Cox risk coefficients and expression values was used as the prognostic risk score, and the median of the risk scores was used to divide the TNBC samples. A log-rank test was then performed between the high-risk and low-risk groups. The prognostic outcomes differed significantly between the high- and low-risk groups (Figure 2G, *p* = 0.00092); the high-risk group corresponded to a relatively poor prognosis. The above results indicate that lncRNAs are involved in promoting acetylation by NAT10.

### 3.3. Screening of Key ac4C-Modified Genes

**Figure 3:**
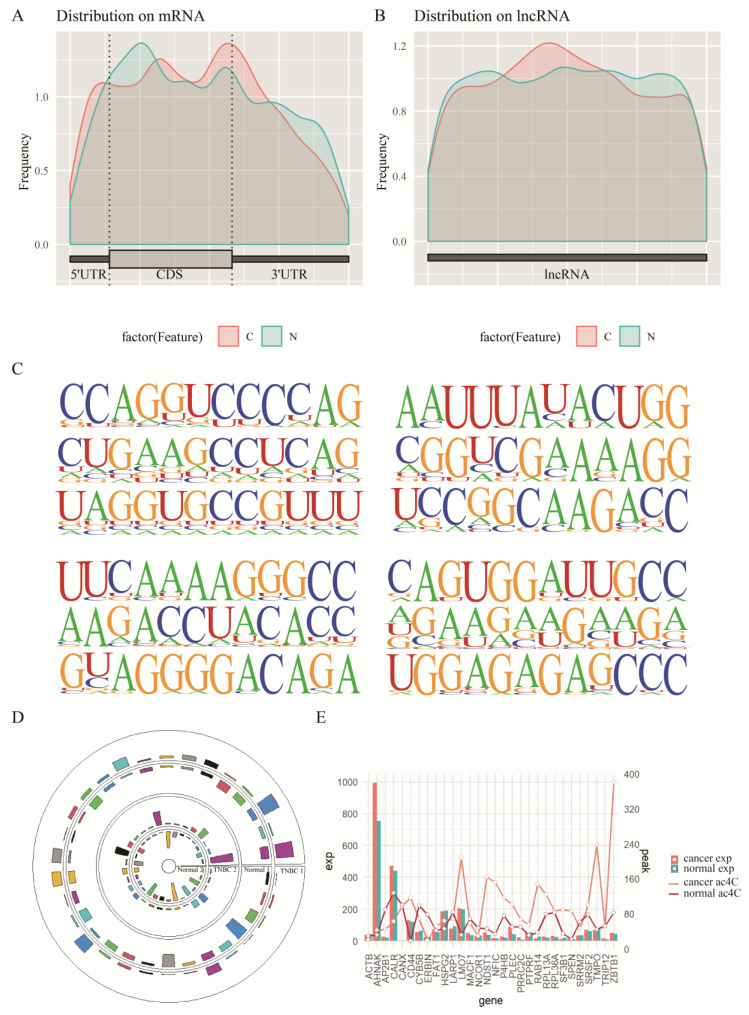
Screening of key ac4C-modified genes. (**A**,**B**): Enriched regions of ac4C modifications in disease and normal groups in twice experiments. (**C**) Significantly enriched motifs of acetylated ac4C modifications in twice sequencing. (**D**) Similar trends in the peak of differential ac4C gene modifications in twice sequencing. (**E**) The change in expression and peak of differential ac4C genes relative to normal samples.

Oberdoerffer from NIH published a study in *Cell*, and showed that a large number of ac4C modifications exist on mRNAs and affect the stability and translation efficiency of mRNAs [30]. To investigate the role of ac4C acetylation in TNBC, the genome-wide acetylation levels of cancer and paracancer tissues from two patients were examined by acRIP-seq. To examine where the peaks were located on the gene structure, all called peaks were annotated and analyzed in this study. Metagene plot maps were drawn based on the total length of the region on the identified ac4C peaks. The cumulative distribution of ac4C modified peaks on RNA structures from both sequencing showed the similar trends, with the enriched regions of ac4C modifications in disease and normal groups mainly concentrated in the CDS region (Figure 3A,B). Peak annotation classifies the structure of the gene to which the peak belongs to 5′UTR, CDS, 3′UTR, and exon. It should be noted that exon refers to an exon of noncoding transcript. Since there are multiple transcripts for a gene, the longest transcript is selected as the annotation type (class). When the class is exon, although the gene type is shown as protein_coding, exon still refers to exon of a non-coding transcript. Peak annotation analysis showed that the enriched regions of ac4C modifications in cancer and paraneoplastic tissues generated by sequencing of tissues from both patients were mainly concentrated in the coding sequence, the 5′-UTR and 3′-UTR regions, with the least distribution in exon regions (Supplementary Figure S1). The enrichment analysis was also performed on the ac4C-modified motifs using HOMER software, and the enrichment of each motif was determined by scanning all sequences. Its significance was calculated by hypergeometric distribution. The top three motif predictions in order of enrichment significance *p* are shown on Figure 3C (*p* < 1 × 10^−15^). The results show that the motifs exhibit a high level of matching with “CXXCXXCXX…” (Figure 3C). Due to the limit of article, the top10 significant motif were shown on Supplementary Figure S2. According to previously published literature [30], the typical form of ac4C modification is “CXXrepeats”, i.e., the motif analysis results in “CXXCXXCXX…”.

The ac4C-seq results obtained from the cancer and paracancer tissues were screened for differential peak ac4C genes separately. The first screen yielded 350 differential ac4C genes, including 174 upregulated and 176 downregulated genes (Supplementary Figure S3). A total of 1242 differential ac4C genes were screened in the second screen, among which there were 858 upregulated and 384 downregulated genes (Supplementary Figure S4). The significantly different ac4C genes from the two tissues were sorted and combined with a difference level of log2FC using RRA tool. The genes with difference significance *p* < 0.05 in the integrated dataset and those differentially modified in both sequencing were selected as the final selection. We eventually obtained 180 differential ac4C genes, of which 30 were significantly different in both patients relative to the normal ac4C-modified peak at the same time, and more than half of the gene-modified peaks showed the same differential trend (Figure 3D). The mean values of these genes relative to the normal modification peaks in both patients as well as the mean expression levels are also shown in Figure 3E. Furthermore, the gene expression data from TCGA and the sequenced gene expression data were corrected for batch effects using COMBAT in R. The gene expression profile of 180 differential ac4C genes in TCGA for TNBC showed the same trend in the expanded sample for the 180 genes (Supplementary Figure S5).

### 3.4. Biological Function of the ac4C Differential Genes in TNBC

**Figure 4:**
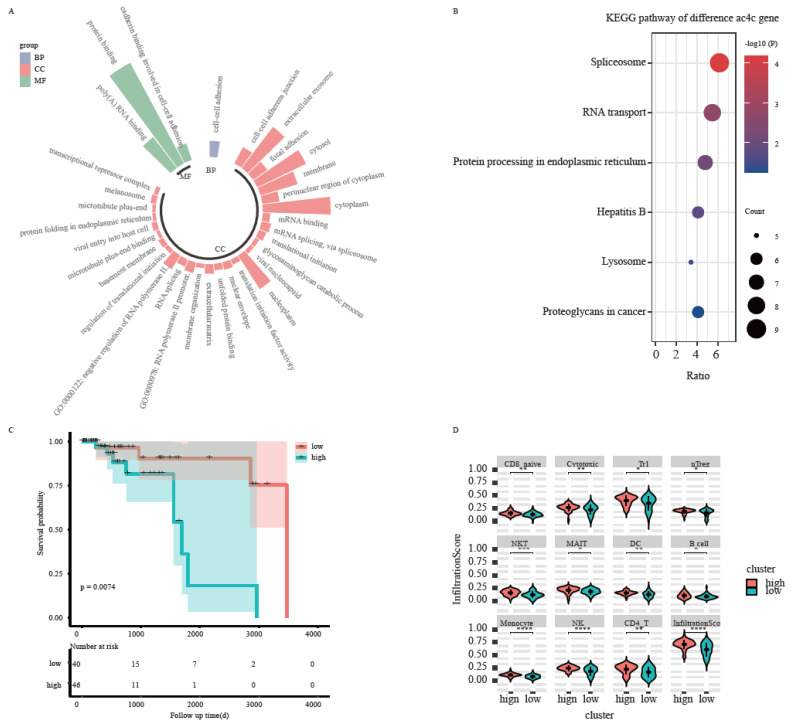
Biological function of the ac4C differential genes. (**A**) Functional enrichment analysis of differential ac4C genes. (**B**) Survival curves of differential ac4C genes. (**C**) Abundance of immune cell infiltration in samples from high-risk and low-risk groups. (**D**): The violin graph of infiltration scores between two groups. *: *p* < 0.05 ; **: *p* < 0.01 ; ***: *p* < 0.001 ; ****: *p* < 0.0001.

To explore the biological functions of the 180 peak differential ac4C genes, GO annotations based on the DAVID database were performed at three levels: BP (biological process), MF (molecular function), and CC (cellular component). Fisher’s exact test was used to calculate the significance level (*p.adj* < 0.01) of each analysis. The results showed enrichment in biological processes such as mRNA transcription–translation processing and, cell–cell adhesion, cellular components such as extracellular exosomes and adherent patches, and molecular functions related to gene expression such as protein binding, RNA binding, and translation (Figure 4A). Pathway annotation of the screened differential ac4C genes was performed based on KEGG, and the significance pathway was calculated using Fisher’s test (*p* < 0.05). The results showed that ac4C-modified altered genes were enriched in pathways such as shear body, RNA transport, lysosome, and hepatitis B (Figure 4B).

Gene expression levels of 180 differential ac4C genes were combined with clinical factors, such as age, gender, stage, and TNM stage of TNBC patients to perform univariate Cox and multivariate Cox regression analyses to find the most prognostically relevant features. Six ac4C genes were identified that were significantly associated with prognosis (*p* < 0.05): *COL3A1*, *CYFIP1*, *SFN*, *SMOC2*, *TRIR,* and *USP8*. Prognostic risk scores were obtained by summing the product of the multivariate Cox coefficients and gene expression values of the six prognostic risk ac4C genes. The median value of the risk scores was used to classify TNBC samples into high- and low-risk groups. The log-rank test was performed between the two groups, and the results showed that the prognosis was significantly different between the high- and low-risk groups (*p* < 0.05) (Figure 4C), and the high-risk group corresponded to a relatively poor prognosis. In addition, using the mean values of the expression levels of the six prognostic risk genes to classify the samples into high-and low-risk groups, the final infiltration Score of all immune cells was shown to differ between the high- and low-risk groups (Figure 4D) (*p* < 0.05).

### 3.5. The ac4C-Related Prognostic Risk lncRNAs in TNBC

**Figure 5:**
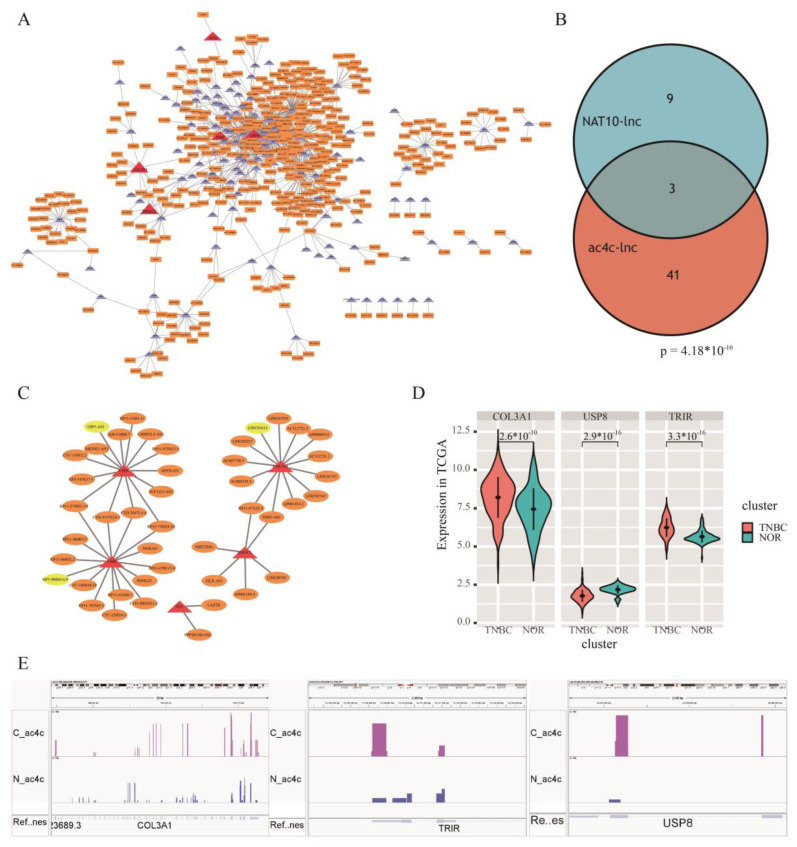
The ac4C-related prognostic risk lncRNAs in TNBC. (**A**) Co-expression network of differentially ac4C genes and differentially expressed lncRNAs. (**B**) Venn of ac4C-related and NAT10-related prognostic risk lncRNAs. (**C**) One-step nearest neighbor co-expression network of key prognostic risk lncRNAs. (**D**) *COL3A1, USP8,* and *TRIR* expression levels in TNBC samples from €A database. (**E**) AcRIP-seq sequencing results of *COL3A1, USP8,* and *TRIR* showed differential acetylation.

To explore lncRNAs specific to TNBC, *t*-tests were performed between TNBC and normal groups in TCGA. This identified 2544 lncRNAs with significant differences in expression (*p.adj* < 0.05). Pearson correlation analysis identified 180 ac4C differential genes and 2544 differentially expressed lncRNAs were screened for significant correlations between them (Pearson, r > 0.5, *p* < 0.05). A co-expression network (Figure 5A) was constructed, and 1080 lncRNA-gene relationship pairs were screened. The results included 436 lncRNAs with differences between TNBC and normal and 116 ac4C differential genes. In particular, five out of six ac4C prognostic risk genes were found in the network, and 44 lncRNAs were co-expressed with them, including three NAT10-influenced prognostic risk lncRNAs: *LINC01614, OIP5-AS1, and RP5-908M14.9* (Figure 5B). The one-step nearest neighbors of the three lncRNAs were extracted from the co-expression network (Figure 5C), and *LINC01614-COL3A1, OIP5-AS1-USP8,* and *RP5-908M14.9-TRIR* showed strong correlation of expression in TNBC. The expression levels of *COL3A1* and *TRIR* showed an upregulated trend in TCGA, but *USP8* showed a downregulation trend (Figure 5D). In addition, all three genes showed differential acetylation as higher in cancer than in paracancerous tissues (Figure 5E). Analysis of the data in the TCGA database also indicated that NAT10 may further regulate ac4C RNAs by regulating the expression of related lncRNAs, thereby affecting the process of acetylation and influencing TNBC prognosis.

### 3.6. Acetylated ac4C Genes Predict Drug Sensitivity in TNBC

**Figure 6:**
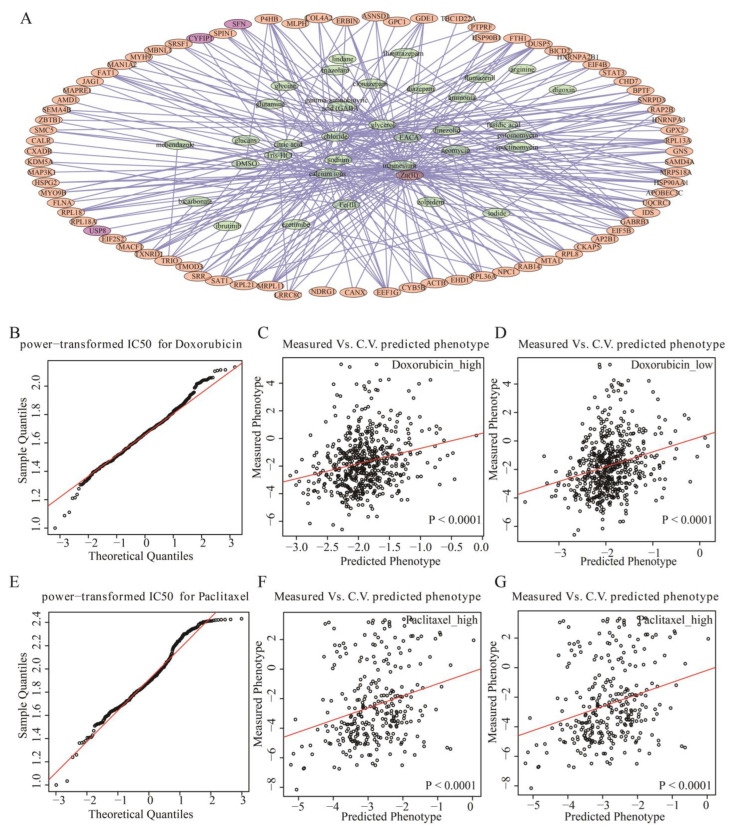
Acetylated ac4C genes predict drug sensitivity. (**A**) Drug-target network of difference ac4C genes. The orange and purple nodes are differential ac4C genes, the green nodes are drug names, and the purple nodes are prognostic risk genes related to NAT10. (**B**) QQ plot of power-transformed IC50 values for doxorubicin. (**C**) Predicted IC50 of doxorubicin in the high-risk group. (**D**) Predicted IC50 of doxorubicin in the low-risk group. (**E**) QQ plot of power-transformed IC50 values for paclitaxel. (**F**) Predicted IC50 of paclitaxel in the high-risk group. (**G**) Predicted IC50 of paclitaxel in the low-risk group.

TNBC is insensitive to endocrine therapy because of ER and PR negativity, and is insensitive to targeted therapy (Herceptin) because of HER2 negativity. The main treatment option is chemotherapy, but the response to conventional chemotherapy is poor. It is important to explore markers that can be used to predict drug sensitivity. To further determine whether differential ac4C genes could help predict effective drug targets in TNBC, 79 of 180 differential ac4C genes were screened for use as drug targets using the DRUGBANK database, which corresponded to 35 drugs. Among them, *USP8*, one of the three prognostic risk ac4C genes obtained from the previous analysis, is a drug target, and corresponded to the drug Zn(II). Zn(II) has an antiproliferative effect on human cancer cells [31] and had stronger active cytotoxicity in inducing morphological changes in breast cancer cell lines compared with cisplatin, and was non-toxic to fibroblasts [32]. The network of differential ac4C genes with drug targets is shown in Figure 6A, in which the orange and purple nodes are 79 differential ac4C genes, the green nodes are drug names, and the purple nodes are prognostic risk genes related to NAT10.

Furthermore, to explore the predictive effect of the three prognostic risk ac4C genes (*USP8*, *COL3A1,* and *TRIR*) regulated by NAT10 on drug-sensitive response, the sum of the product of the Cox risk coefficients of the three genes and the expression values were used as the prognostic risk score, and the median value of the risk scores was used to divide TNBC samples into high- and low-risk groups. Drug sensitivity prediction was performed using the R package “pRRophetic”. Drugs commonly used in TNBC are doxorubicin and paclitaxel. (Figure 6B,E), and their IC50 data were obtained from the GDSC database. To verify whether the drugs met the linear model, a QQ plot was produced and showed that both drugs were in line, and the linear model criteria could be used to predict the IC50 values of the samples using ridge regression. Next, ridge regression was used to estimate the half-maximal inhibitory concentration (IC50) of the TCGA samples and tenfold cross-validation was used to assess the predictive accuracy of the estimated IC50. The Mann–Whitney–Wilcoxon test was used to test whether the IC50 distribution was the same for the high-risk and low-risk groups.

Doxorubicin, a highly effective broad-spectrum anticancer drug widely used in TNBC, is an anthracycline antibiotic that embeds between the DNA double helix and inhibits replication after strand unwinding. It significantly predicted drug sensitivity in both high- and low-risk groups, allowing particularly accurate estimation of IC50 values in patients (Figure 6C,D). Paclitaxel inhibits microtubule depolymerization by inhibiting mitosis in cancer cells, and the PD-L1 antibody, atezolizumab, in combination with albumin–paclitaxel was approved by the FDA as standard therapy for PD-L1-positive TNBC. In TNBC samples from TCGA, the sensitivity of the drug was significantly predicted in both high- and low-risk groups classified by prognostic risk ac4C genes regulated by NAT10, and the IC50 value could be accurately estimated (Figure 6F,G). However, there was no significant difference in sensitivity between the two drugs in the high- and low-risk groups (Supplementary Figure S6), which also demonstrated the high-risk of TNBC and the lack of effective targeted drugs. In particular, lncRNAs (*LINC01614*, *OIP5-AS1,* and *RP5-908M14.9*) interacting with three prognostic risk ac4C genes can also significantly predict drug sensitivity in TNBC patients, but with low relative accuracy (Supplementary Figure S7).

## 4. Discussion

Acetylcytidine is an ancient, evolutionarily conserved modification. There is increasing evidence to support a strong link between acetylation imbalance and carcinogenesis. Mutations in the gene encoding acetyltransferase (HAT) lead to the development of various solid tumors, such as colorectal cancer [33] and gastric cancer [34]. In this study, acRIP-seq technology was used to detect genome-wide acetylation levels in cancer and paracancerous tissues of two patients. We identified 180 shared differentially acetylated genes, which showed similar expression differences in TNBC samples from TCGA. The functions of these ac4C genes are significantly enriched in biological processes (mRNA transcription, translation, and intercellular adhesion) and molecular functions related to gene expression (protein binding, RNA binding, and translation). Among them, intercellular adhesion is significantly associated with cancer metastasis [35]. Combined with TCGA expression and clinical data, 6 ac4C genes (*p* < 0.05) significantly associated with prognosis were screened out of 180 differentially acetylation genes, namely, “*COL3A1*”, “*CYFIP1*”, “*SFN*”, “*SMOC2*”, “*TRIR*”, and “*USP8*”. Their expression levels significantly affected the survival time of TNBC patients.

NAT10 is an acetyltransferase belonging to the GCN5-associated N-acetyltransferase (GNAT) family [35]. It can acetylate target proteins, regulate the DNA damage response [36] and affect cancer development [37]. NAT10 catalyzes N4-acetylcytidine (ac4C) deposition on different RNA substrates and is involved in colon cancer invasion and metastasis [38]. lncRNAs can be activated by acetylation and act as miRNA sponges in retinoblastoma to activate signaling pathways and induce cancer [39]. In this study, to explore the sponge function of acetylation genes in TNBC, 2544 lncRNAs with significant differences in expression between TNBC and normal groups were screened from the TCGA database. Using Pearson correlation analysis, a co-expression network of 180 ac4C differential genes and 2544 differentially expressed lncRNAs was constructed and included 1080 lncRNA-gene relationship pairs. The three lncRNAs in the co-expression network were prognostic risk lncRNAs affected by NAT10, namely, “*LINC01614*”, “*OIP5-AS1*”, and “*RP5-908M14.9*”. Their interaction pairs in the network were *LINC01614—COL3A1*, *OIP5-AS1—USP8*, and *RP5-908M14.9—TRIR*. *COL3A1* has been significantly associated with breast cancer brain metastases in multiple studies [40,41]. The inhibition of ubiquitin-specific protease 8 (USP8), a novel deubiquitylase of the Notch1 intracellular domain, downregulated the Notch signal pathway, resulting in the retardation of cell growth and colony forming ability of breast cancer cell lines [42]. The relevance of USP8 for patients with BRCA has been reported, with high expression correlating with better clinical features [43]. There have been few reports addressing the role of TRIR in breast cancer. However, in melanoma, TRIR inhibits angiogenesis and is related to the activity of cancer cells [44]. This study found that NAT10-related acetylation genes play an important role in cancer, but the molecular mechanism of TNBC has not been fully confirmed. In the future, these genes can be used as potential therapeutic targets to predict the survival time of TNBC patients and to develop effective treatments.

Because TNBC is negative for ER, PR, and HER2, it is not sensitive to endocrine therapy or targeted therapy. We selected doxorubicin and paclitaxel, which are commonly used in TNBC, and downloaded the sensitivity data of these two drugs from the GDSC database. Three prognostic risk ac4C genes (*USP8*, *COL3A1,* and *TRIR*), identified in this study and regulated by NAT10, were used to predict drug sensitivity. In the high- and low-risk groups, their expression levels can significantly predict drug sensitivity and accurately estimate IC50. However, there is no significant difference in the sensitivity of the two drugs in the high- and low-risk groups, which also proves that there is a lack of effective drugs for the treatment of TNBC patients. Therefore, TNBC-targeted drugs are urgently needed. A small molecule inhibitor of NAT10 remodeling was discovered in 2014, and can repair nuclear defects and improve chromatin structure in lamellar cells and progeria diseases by inhibiting NAT10 [45]. Therefore, it is highly desirable to develop a NAT10-based drug for the treatment of cancer. To explore potential drug targets for TNBC, relevant data of drugs and drug targets were obtained from the DRUGBANK database, and they were intersected with 180 ac4C differential genes. The results showed that 79 of the 180 differential ac4C genes were drug targets, corresponding to 35 drugs. Among them was the prognostic risk ac4C differential gene, USP8, and its corresponding drug is Zn(II). Zinc (Zn) provides structural integrity for many proteins, such as in zinc finger proteins. The National Cancer Institute (NCI) found that certain tumor cell groups are sensitive to specific metal agents. Zn complex compounds can induce adaptive tumor immunity to cancer cells [46]. Zn(II) arginine dithiocarbamate complex is actively cytotoxic; however, compared with cisplatin, it has lower cytotoxicity in inducing morphological changes in the T47D breast cancer cell line [32]. This indicates the feasibility of Zn(II) as a targeted drug for TNBC cancer.

## 5. Conclusions

This study investigated NAT10, which promotes RNA ac4C acetylation, combined with acRIP-seq technology, to examine lncRNAs to explore the ac4C genes in depth that significantly affect the prognosis of TNBC patients. These results showed that RNA ac4C acetylation can significantly affect the prognosis of TNBC and can accurately predict its sensitivity to chemotherapeutic drugs through the involvement of lncRNAs. The predicted drug targets based on RNA ac4C acetylation are credible and will aid the development of targeted drugs. However, the specific mechanism of RNA ac4C acetylation needs to be elucidated.

## Data Availability

The data that support the findings of this study are available from the corresponding author upon reasonable request. The acRIP-seq data of TNBC samples supporting this study are available from China National Center for Bioinformation/Beijing Institute of Genomics, Chinese Academy of Sciences (https://ngdc.cncb.ac.cn (accessed on 10 July 2022), PRJCA012215).

## Supplementary Materials

The following supporting information can be downloaded at: https://www.mdpi.com/article/10.3390/genes13122400/s1, Figure S1. Peak annotation analysis of ac4C modification-enriched regions in cancer and primary paraneoplastic tissues in sequencing results. Figure S2. Top 10 significant motifs performed by peaks calling from second sequencing. Figure S3. Volcano plot of differential ac4C genes obtained from the first samples. Figure S4. Volcano plots of differential ac4C genes obtained from the second samples. Figure S5. Expression of differential ac4C gene in TCGA. Figure S6. Sensitivity to doxorubicin and paclitaxel in high- and low-risk groups classified by prognostic risk ac4C genes regulated by NAT10 protein. A. The boxplot of Doxorubicin IC50 in low- and high-risk samples. B. The boxplot of Paclitaxel IC50 in low- and high-risk samples. Figure S7. Association of lncRNAs interacting with the prognostic risk ac4C gene and drug sensitivity in TNBC patients. A. The association plot of measured vs. predicted phenotype in Doxorubicin_high group. B. The association plot of measured vs. predicted phenotype in Doxorubicin_low group. C. The association plot of measured vs. predicted phenotype in Paclitaxel_high group. D. The association plot of measured vs. predicted phenotype in Paclitaxel_high group.
