# Supplementary material for: Revealing the Potential Markers of N(4)-Acetylcytidine through acRIP-seq in Triple-Negative Breast Cancer
Source: Genes (Basel). 2022 Dec 18;13(12):2400. doi: 10.3390/genes13122400 (PMC9777589; doi:10.3390/genes13122400)

Supplementary Figure S1

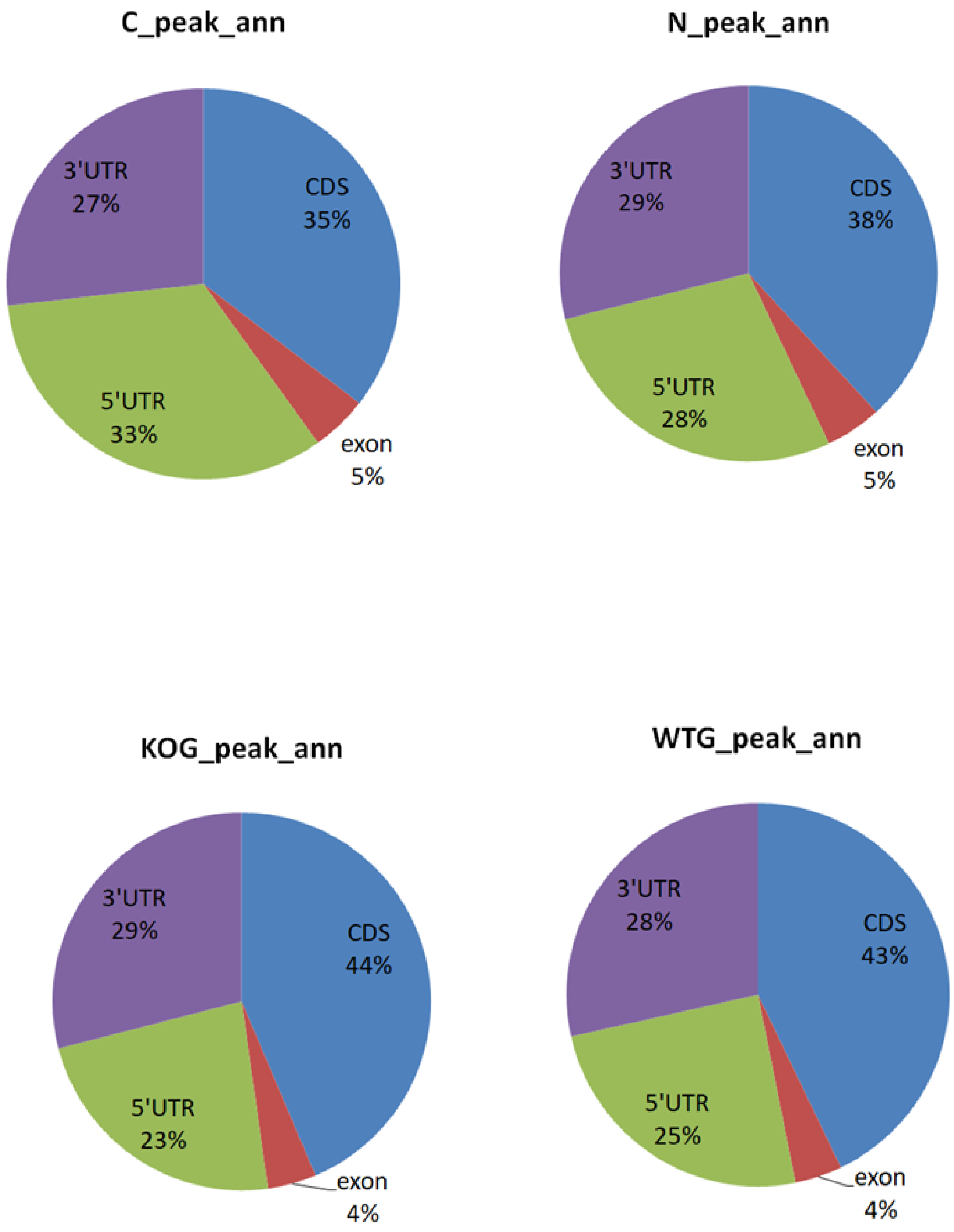

Supplementary Figure S2

Motif Results

A

Caner_homer

B

WTG_homer

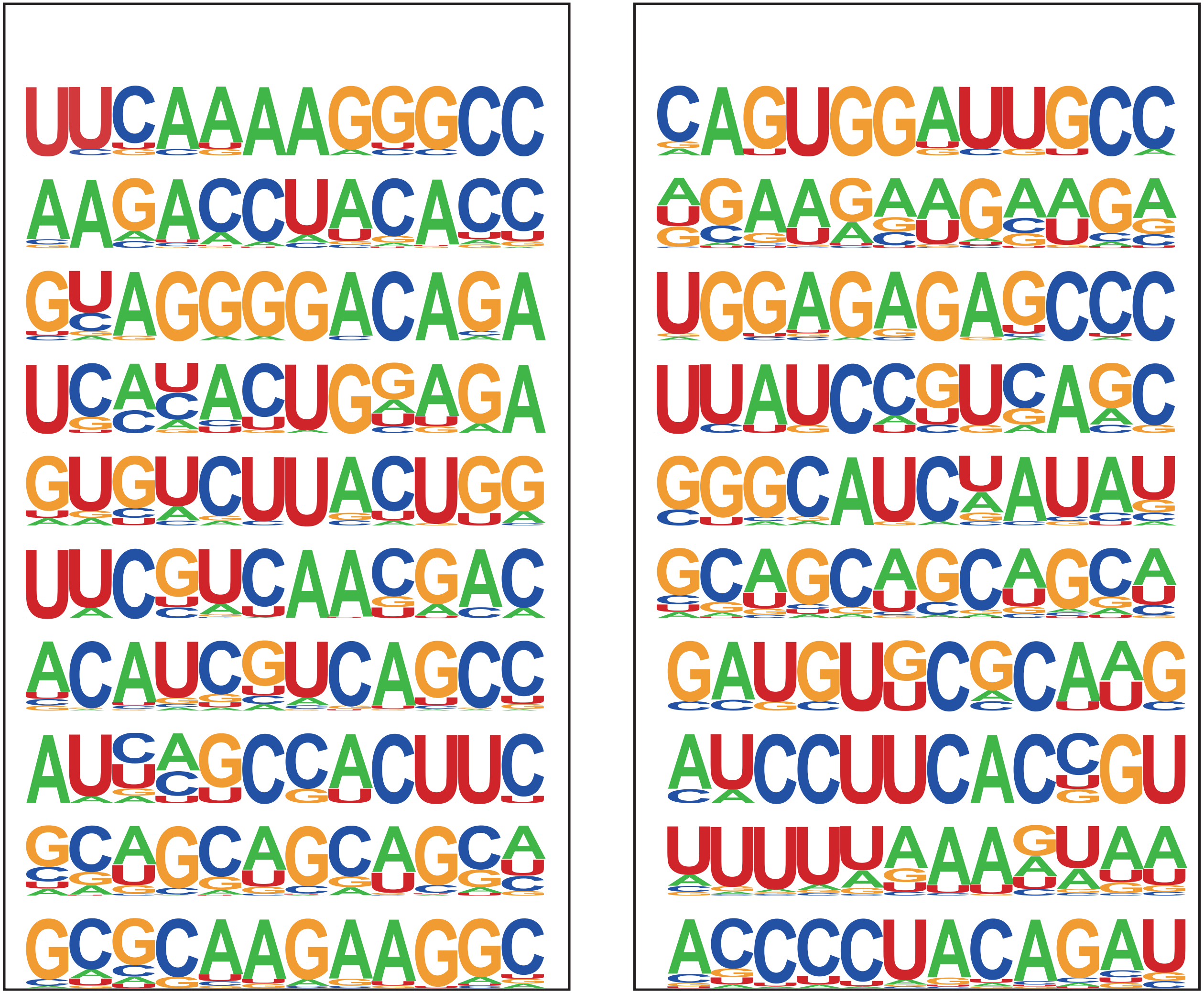

Supplementary Figure S3

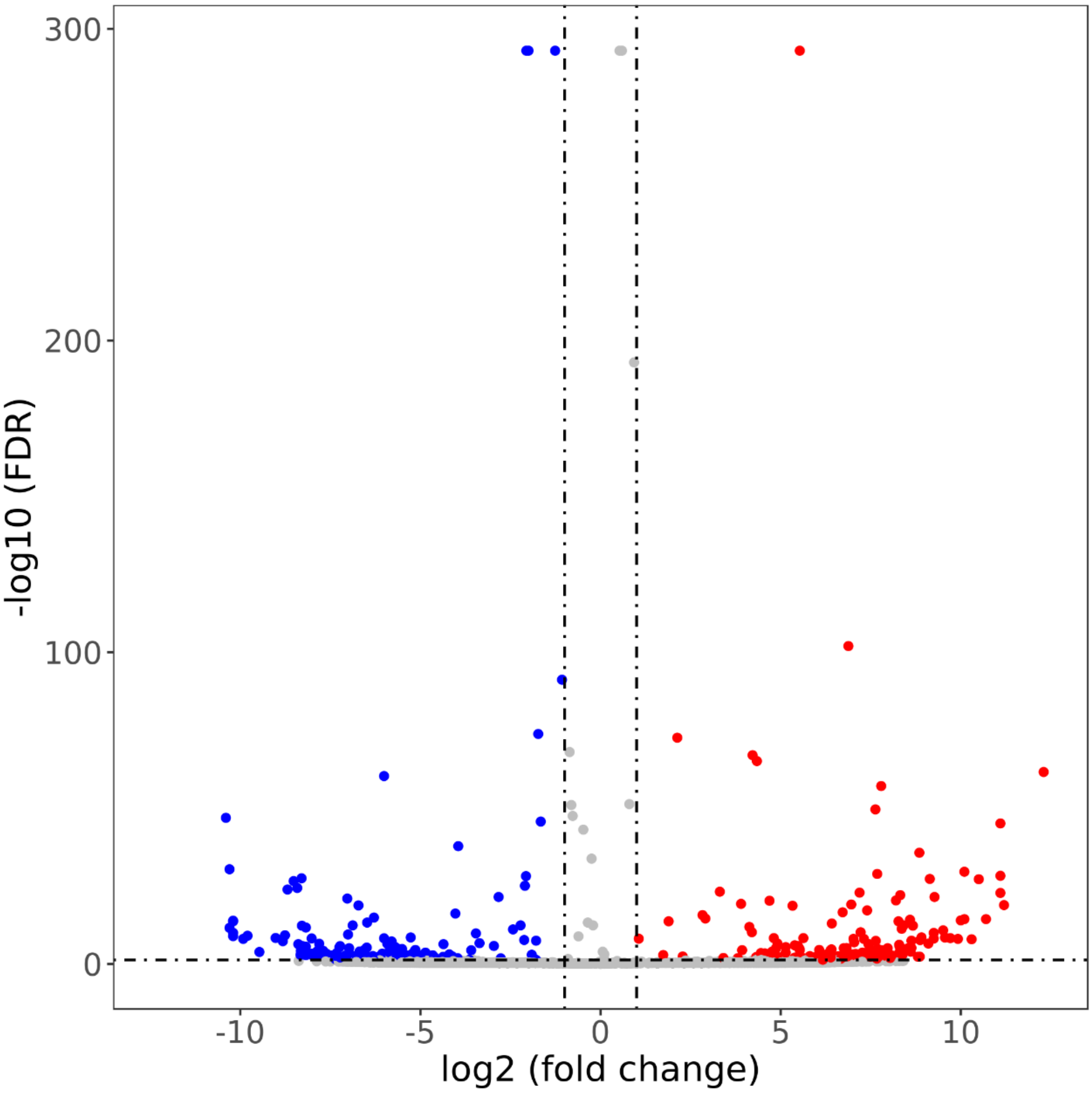

Supplementary Figure S4

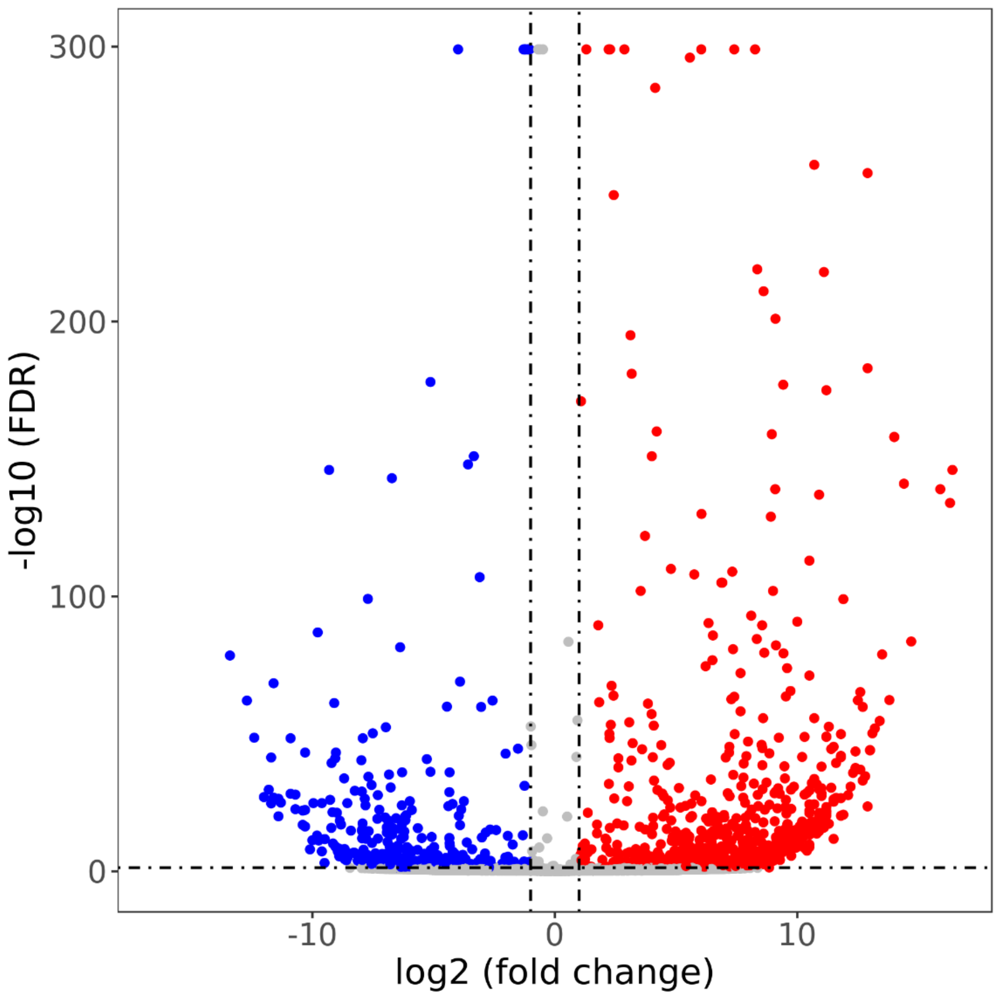

Supplementary Figure S5

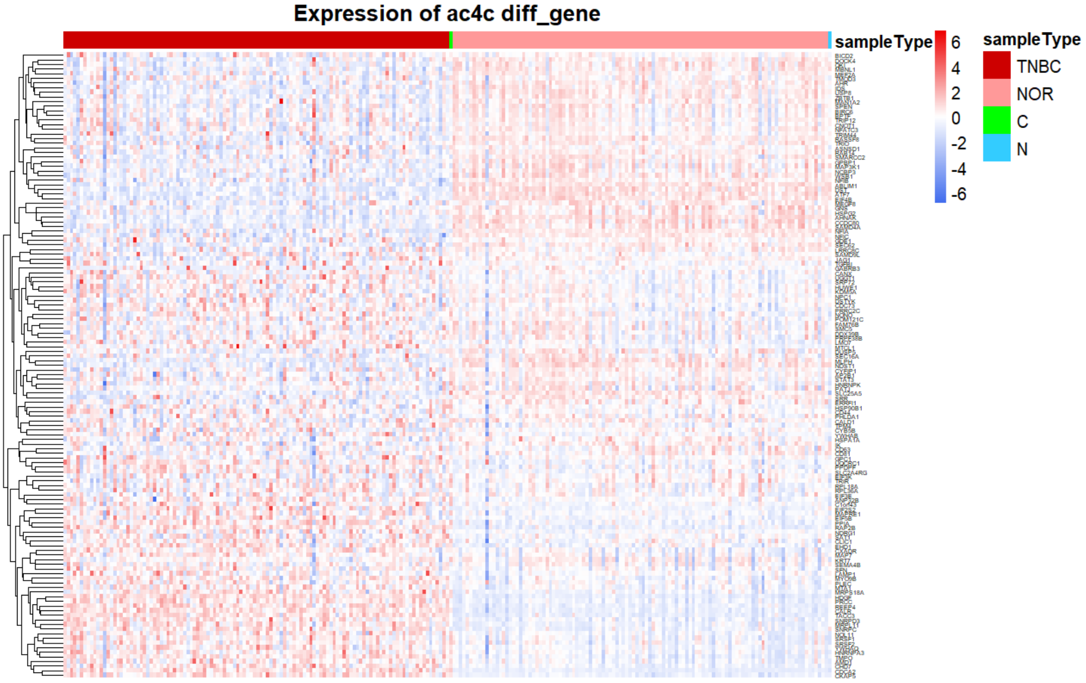

Supplementary Figure S6

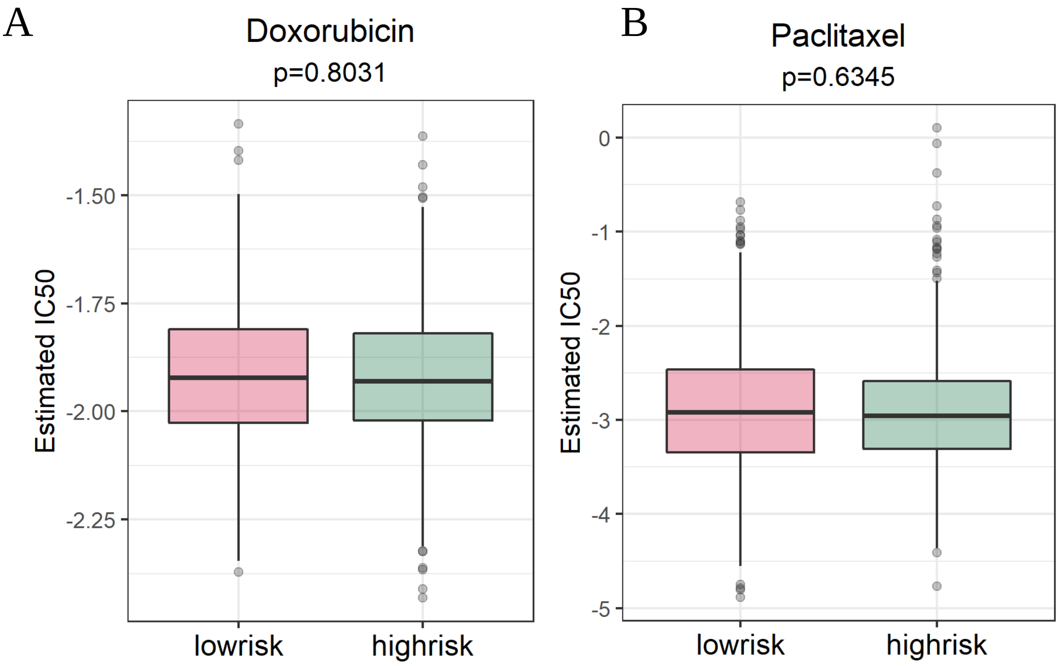

Supplementary Figure S7

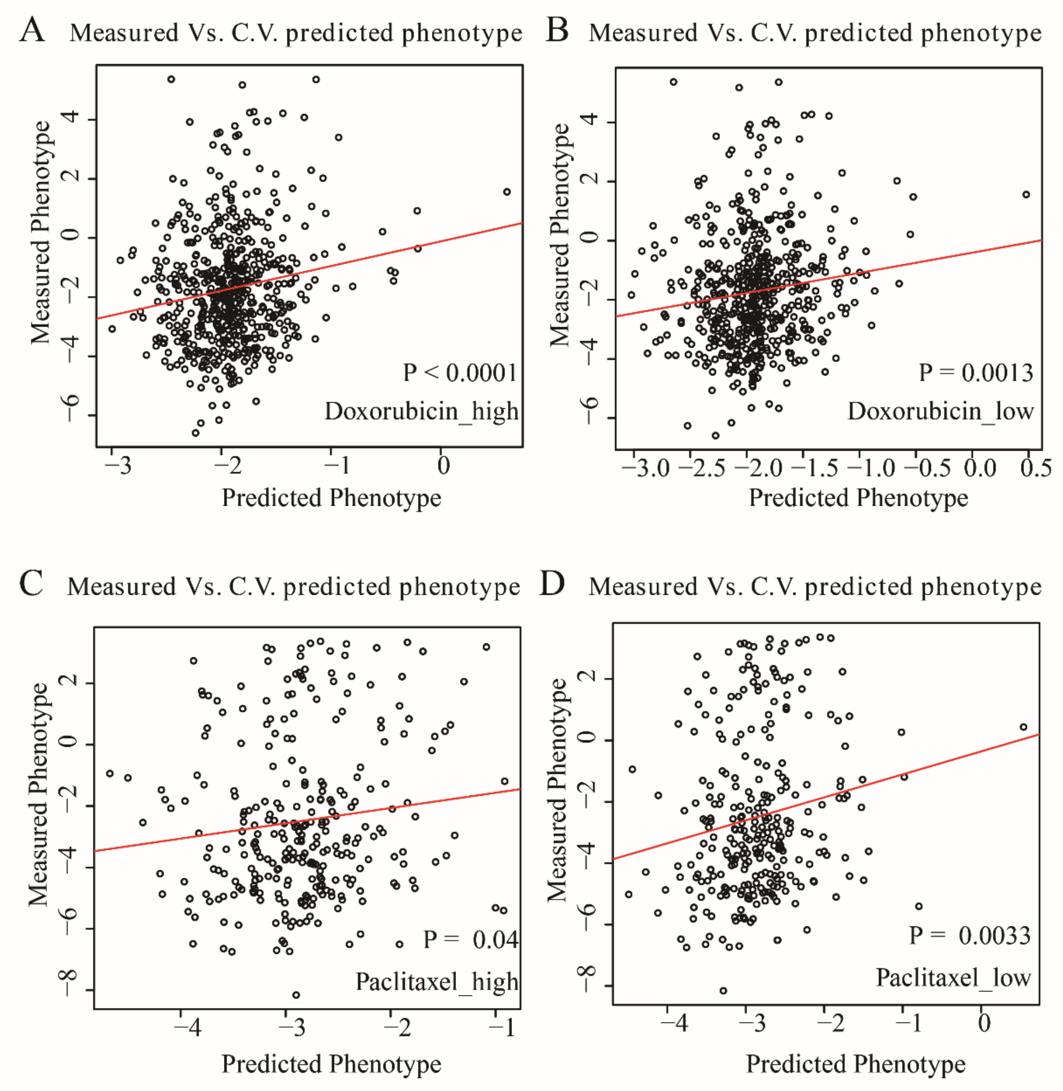

Supplement: Supplementary file 1 [file genes-13-02400-s001.zip › genes-2028406-supplementary.pdf]
